# *In Silico* Prediction of Estrogen Receptor Subtype Binding Affinity and Selectivity Using Statistical Methods and Molecular Docking with 2-Arylnaphthalenes and 2-Arylquinolines

**DOI:** 10.3390/ijms11093434

**Published:** 2010-09-20

**Authors:** Zhizhong Wang, Yan Li, Chunzhi Ai, Yonghua Wang

**Affiliations:** 1 Center of Bioinformatics, Northwest A&F University, Yangling, Shaanxi, China; E-Mail: wzhizhong@nwsuaf.edu.cn; 2 School of Chemical Engineering, Dalian University of Technology, Dalian, Liaoning, China; E-Mail: adinalee@163.com; 3 Pharmaceutical Resource Discovery, Dalian Institute of Chemical Physics, Chinese Academy of Sciences, Dalian, Liaoning, China; E-Mail: aicy@dicp.ac.cn

**Keywords:** receptor, selectivity, QSAR, docking

## Abstract

Over the years development of selective estrogen receptor (ER) ligands has been of great concern to researchers involved in the chemistry and pharmacology of anticancer drugs, resulting in numerous synthesized selective ER subtype inhibitors. In this work, a data set of 82 ER ligands with ERα and ERβ inhibitory activities was built, and quantitative structure-activity relationship (QSAR) methods based on the two linear (multiple linear regression, MLR, partial least squares regression, PLSR) and a nonlinear statistical method (Bayesian regularized neural network, BRNN) were applied to investigate the potential relationship of molecular structural features related to the activity and selectivity of these ligands. For ERα and ERβ, the performances of the MLR and PLSR models are superior to the BRNN model, giving more reasonable statistical properties (ERα: for MLR, R_tr_^2^ = 0.72, Q_te_^2^ = 0.63; for PLSR, R_tr_^2^ = 0.92, Q_te_^2^ = 0.84. ERβ: for MLR, R_tr_^2^ = 0.75, Q_te_^2^ = 0.75; for PLSR, R_tr_^2^ = 0.98, Q_te_^2^ = 0.80). The MLR method is also more powerful than other two methods for generating the subtype selectivity models, resulting in R_tr_^2^ = 0.74 and Q_te_^2^ = 0.80. In addition, the molecular docking method was also used to explore the possible binding modes of the ligands and a relationship between the 3D-binding modes and the 2D-molecular structural features of ligands was further explored. The results show that the binding affinity strength for both ERα and ERβ is more correlated with the atom fragment type, polarity, electronegativites and hydrophobicity. The substitutent in position 8 of the naphthalene or the quinoline plane and the space orientation of these two planes contribute the most to the subtype selectivity on the basis of similar hydrogen bond interactions between binding ligands and both ER subtypes. The QSAR models built together with the docking procedure should be of great advantage for screening and designing ER ligands with improved affinity and subtype selectivity property.

## 1. Introduction

The estrogen receptor (ER), a member of the nuclear receptor superfamily of ligand-modulated transcriptional factors [1], is responsible for transcription of genes containing estrogen responsive elements or repression of some genes [2]. ER mediates the activity of estrogens in the regulation of a number of important physioligical processes, including the development and function of the female reproductive system and maintenance of bone mineral density and cardiovascular health; however stimulation of other tissues can increase the risk of cancer within these tissues, particular in female breast and uterus [3]. Thus, ER has been a target for pharmaceutical agents for hormone replacement in menopausal women, uterine and breast cancers.

ER was found in two isoform subtypes, *i.e.*, ERα and ERβ. Studies have shown the two subtypes have different functions and distributions in certain tissues [4,5]. Molecules that selectively activate

ERβ not only hold promise for the treatment of certain cancers, endometriosis and inflammatory diseases and cardiovascular and CNS conditions [6], but also have a profound effect in regulating brain development and estrogen-induced promotion of neurogenesis and memory, in conjunction with its reduced feminizing effects [7]. In addition, there are unexpected adverse effects of the ER ligands already used as clinical agents. Extensive efforts are being made to develop subtype-specific ligands which selectively antagonize undesirable estrogenic effects, while promoting positive estrogen effects for therapeutic purposes.

The mobility and plasticity of the ER ligand binding domain (LBD) allow compounds of extraordinary structural diversity mimicking natural estrogen agonists or antagonist to bind to ER subtypes. Remarkably, the smaller LBD volume for ERβ comparing to that for ERα and differences in the amino acids hold promise of discover and design ligands with a degree of subtype-selective agonist/antagonist character. Nevertheless, the similarity of the binding pocket between ERα and ERβ increases the difficulty of developing ligands having sufficient levels of ERβ selectivity and binding affinity. The key issue in the design of new selective ER ligands is to explore the properties of the chemical structure in combination with its ability of inducing a pharmacological response as a consequence of receptor-binding. Great advances have been made in recent years because of multiple structurally diverse compounds were synthesized and have been shown to exhibit unprecedented estrogen receptor subtype selectivity [8–11].

Most of these synthesized compounds are based on the scaffolds of the known ER subtype selective compounds. However, this cannot avoid the risk that non selective or low binding affinity compounds will be synthesized and tested experimental, which may result in a tremendous financial cost and waste of time. Thus, the need for rapid and cost-effective screening tools to detect and characterize the agents with selective ER subtype binding affinity is urgent. Since compounds already confirmed by experimental assays provide an opportunity to understand the basis of subtype selectivity, the development of models for predicting the subtype selectivity would allow for the development of more potent and selective compounds for these important pharmaceutical targets. In this context, QSARs can be of valuable assistance in predicting the estrogenic activity of certain molecules [12].

Numerous QSARs have been developed to predict hormone relative binding affinity and to indicate potential estrogenicty, such as CoMFA [13], KNN [14], HQSAR [15]. However, for studying the structural information relating to the binding affinity for ERα and ERβ, finding the subtype selective ligands with proper binding affinity, counTable QSAR models are available. Peter [16] constructed CoMFA models to both isoforms of the ERs. When validated by the most predictive models, the most selective ligands were ranked correctly. ANNs (artificial neural networks) were used to model selective binding of 48 phytoestrogens and structurally related compounds at ERα and ERβ by Agatonovic-Kustrin [17] and some structural characteristics responsible for the selective binding to ERα and ERβ were identified. Barrett *et al*. [18] synthesized a group of benzoxepin-derived ER ligands and investigated the subtype selectivity using a PLS model combining different descriptors with the endpoint LogIC_50_ (ER*β/α*).

3D-QSAR techniques are generally considered to be the most effective means of predicting biological activity. However, they usually require an accurate superposition of structures, which has proven to be the major bottleneck [19,20]. Classical linear QSAR methods relay on a higher number and better quality of molecular descriptors that cover a broader range of structural characteristics, providing an alternative perspective on the ligand binding properties of the ERs that might be important for the activity [21]. Compared with the linear QSAR models, Bayesian regularized neural networks (BRNNs) have the advantage of managing data containing non-linear relationships for modeling and predictive purpose avoiding the overtraining and overfitting problems that perplex the NN (neural network) applications in generating QSAR models, compared with conventional networks [22,23].

The aim of this paper was to investigate the structural features contributing to the binding affinity of a series of 2-arylnaphthalene and 2-arylquinoline derivatives to ERα and ERβ receptors. More importantly, we are very interested in investigating the structural characteristics contributing to the subtype selectivity profile and to try to discover new selective ERβ-agonists with proper binding affinity. To this end, MLR and PLS regression (PLSR), in combination with a Bayesian method, *i.e.*, BRNN, were used for the investigation. In addition, as an alternative and supplemental approach to QSAR methods, Surflex-Docking procedure was undertaken, which shed further light on the QSAR models built and searching the putative binding modes for the screening purpose. This should be useful for guiding future medicinal chemistry efforts designed to discover selective ligands of ERβ having increased binding affinity and higher selectivity.

## 2. Material and Methods

### 2.1. The Data Set

The data set used in the investigation contains 82 ER ligands, mainly represented by 2-arylnaphthalene and 2-arylquinoline derivatives, which were collected from the literature [9,24]. These compounds were designed specifically to mimic the genistein framework producing new ER ligands with improved binding affinity. The affinity as measured by IC_50_s for human ERα or ERβ of all the compounds was determined by a competitive radioligand binding assay [9,24]. For QSAR analysis, negative logarithm of IC_50_ values, *i.e.*, pIC_50_ (M), were generated. Further, molecular descriptors correlating with the selectivity (*S*) of binding affinity of ligands between ERα and ERβ were investigated, which can greatly beneficial the modulator screen and drug design. Herein we developed the following equation at the premise of the αIC50 is larger than βIC50 of the ligands:

(1) $$S={log}_{10}\left(\frac{\alpha IC50-\beta IC50}{\beta IC50}\right)$$

where a high *S* value indicates a priority to bind the LBD of ERβ. The *S* value increases, the selectivity power between the two ER subtypes increases, and when *S* > 1, corresponding ligands have, at least, a 10-fold binding affinity with ERβ than ERα and are recommended for the SERM screen process. Detailed information of the compounds in the data set (SIMLE strings, corresponding pIC_50_ values for both ERα and ERβ, the S values) is presented in Table 1 as supplementary information.

### 2.2. Molecular Descriptors

The molecular descriptors were calculated with the DRAGON program packages which were originally developed by the Milano Chemometrics and QSAR Research Group (www.disat.unimib.it/chm/). DRAGON provides more than 1,600 molecular descriptors that are divided into 20 logical blocks, which contain not only the simplest atom type, functional group and fragment counts, but also several topological and geometrical descriptors. Some molecular properties such as logP, molar refractivity, and number of rotaTable bonds, H-donors, H-acceptors, and topological surface area (TPSA) are also calculated. According to the energy minimized 3D conformation of each compound, 1,664 2D and 3D molecular descriptors were computed with DRAGON packages based on the structure of a compound. Constant or near constant values and descriptors with zero standard deviations were excluded in order to reduce redundant and non useful information. Finally 1,333 DRAGON descriptors were retained.

### 2.3. Statistical Methods

For data analysis and modeling, multiple Linear Regression (MLR), partial least squares regression (PLSR) and Bayesian regularized neural network (BRNN) investigations were performed. MLR attempts to model the relationship between two or more explanatory variables and a response variable by fitting a linear equation to the observed was employed to correlate the binding affinity and molecular descriptors. This method has been widely applied in many QSAR studies, and has proven to be a useful linear regression method to build QSAR models that may explore straightforward the properties of the chemical structure in combination with its ability of inducing a pharmacological response [25]. In the procedure, stepwise method was introduced to extract the most correlate descriptors.

PLSR is a statistical method that bears some relation to principal components regression; instead of finding hyperplanes of maximum variance between the response and independent variables, it finds a linear regression model by projecting the predicted variables and the observable variables to a new space and is used to find the fundamental relations between two matrices (X and Y), *i.e.*, a latent variable approach to modeling the covariance structures in these two spaces. A PLS model will try to find the multidimensional direction in the X space that explains the maximum multidimensional variance direction in the Y space. PLS-regression is particularly suited when the matrix of predictors has more variables than observations, and when there is multi-collinearity among X values. The detailed algorithm of this method can refer [26,27].

Backpropagated artificial neural networks (ANNs) have been widely used for molecular modeling due to their computational efficiency and their ability for approximating any mapping between independent and response variables. However, their inherent unstability and the existence of overfitted solutions increase when the number of parameters is increased [28]. The Bayesian regularization overcomes the deficiencies of ANNs by modifying the ANNs performance. The Bayesian framework deals with uncertainly by applying probabilities to each possible event [29,30]. In contrast to conventional network training, where an optimal set of weight is chosen by minimizing an error function, Bayesion approach involves a probability distribution of the network weight. After the data is taken, the density function for the weights can be updated according to Bayes’ rule:

(2) $$P(\omega |D,\alpha ,\beta ,M)=\frac{P(D|\omega ,\beta ,M)P(\omega |\alpha ,M)}{P(D|\alpha ,\beta ,M)}$$

where D is the data set, M is the particular neural network model used and ω is the vector of net work weights. *P*(*ω|D*,*α*,*β*,*M*) is the posterior probability, that is the plausibility of a weight distribution considering the information of the data set used. *P*(*ω|D*,*α*,*M*) is the prior density, which represents our knowledge of the weights before any data are collected. *P*(*D|ω*,*β*,*M*) is the probability of the data occurring, given the weights. *P*(*D|α*,*β*,*M*) is a normalization factor, which guarantees that the total probability is 1. Gauss-Newton approximation [31] to Hessian matrix of the objective function *F*(*ω*) has been developed to effectively calculate the regularization. Bayesian methods produce predictors that are robust and well matched to the data which make optimal predictions.

In this work, two-layer networks were fully connected, with a hyperbolic tangent function employed in the hidden layer and a linear transfer function in the output layer. The Levenberg-Marquardt training algorithm [32] was introduced to accelerate the convergence of the targets. The starting-values for the BRNN model parameters were selected according to Nguyen-Widrow rules [33]. The training is stopped at the maximum of the evidence for the hyperparameters α and β [34].

### 2.4. Construction of Training and Test Set

As external validation can provide a more rigorous evaluation of a model's predictive capability for untested chemicals, the best proof of an already developed model’s accuracy is to test model performance on these additional data. For this purpose, before the models were built the whole data set was split into two subsets, *i.e.*, the training set used to build the model and the independent test set to validate the model's accuracy. In this investigation, we performed this splitting on the basis of their distribution in the chemical space which is defined by the Kohonen neural network [35].

A self-organizing map (SOM) creates a set of prototype vectors representing the dataset and carries out a topology preserving projection of the prototypes form the *d*-dimensional grid. This grid is a convenient visualization space for showing the cluster structure of the data. Thus, similar objects were mapped into the same position(x, y coordinates in a Kohonen map). In this work, only a part of representative object form each position in the map was chosen for the training set, respecting the original proportion among the different classes.

### 2.5. Docking

Structurally, the C-terminal LBD of the ERs forms a 3D wedge-shaped binding pocket composed of non-polar residues in the active site, resulting in a largely hydrophobic pocket. This pocket displays specific binding features, allowing it to accommodate a varied set of steroid-like ligands. Basically, we believe that a compound enters the active site of ER in a penetration manner, since this pocket is a narrow-long channel with different hydrophobicity in the two terminals of the channel. Therefore, a hydrophobic compound with different structural ends revealing the hydrophobic variations can easily penetrate into the pocket and bind to a hydrophobic area in the protein [36]. The mechanism might explain the binding characteristics of most ligands in the ER ligand binding domain. In this work, in order to probe the possible binding conformations of ligands in the ER LBD and further rationalize ER subtype selectivity of these compounds, a molecular docking method was also employed.

Surflex-Dock docks ligands automatically into a receptor’s ligand binding cavity using a protomol-based method and an empirically derived scoring function. The protomol is a unique and important factor of the docking algorithm and is a computational representation of assumed ligands that interact with the binding cavity. In addition to the automated docking process, the function in Surflex-Dock has been improved by incorporating a base portion matching algorithm that allows a fragment of the ligand to be prepositioned as it docks in the binding site. The scoring function based on the binding affinities of protein-ligand complexes and on their X-ray structures contains hydrophobic, polar, repulsive, entropic and solvation terms [37,38]. The Cscore functions are also available in the Sybyl software package.

Crystal structures of human ERα and ERβ with same ligands co-crystallized can enhance the accuracy when comparing a ligand docking poses in ERα and ERβ. In this work, six ligand-co-crystallized ER structures were used and the X-ray crystallographic data were retrieved from the Protein Data Bank (PDB ID 1X7R and 1X7E for ERα; 1QKM, 1X78, 1YYE and 1YY4 for ERβ). As listed in Table 2, 1X7R and 1QKM, 1X7E and 1X78 have the same co-crystallized ligands. 1YYE and 1YY4 were chosen because the co-crystallized ligands are also within the studied compound sets (compound6 and compound23).

Prior to docking, in the protein preparation procedure all waters were removed and the hydrogen atoms were added in predicted models using the Biopolymer module in a random way. Protomol for Surflex-Dock was generated according to the software protocol. Two important factors bloat and threshold that can significantly affect the size and extent of the protomol were adjusted in order to get the best docking results. For 1X7R, 1QKM, 1X78, 1YYE and 1YY4, bloat was set to 0.0 and threshold was set to 0.50. For 1X7E, these parameters were set to 0.0 (bloat) and 0.70 (threshold). Before employed to the docking stimulation, all the ligands were energetically minimized employing the Tripos force field and Gasteiger-Huckel charges. Besides, other parameters with default setting and Cscore functions were employed in all runs.

## 3. Results

**Figure 1:**
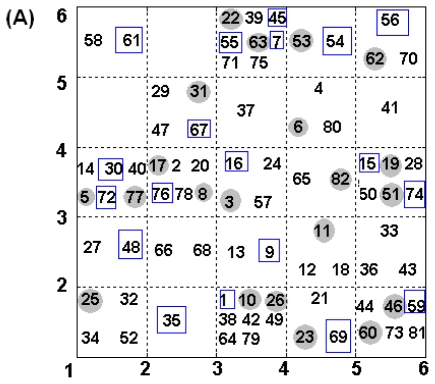

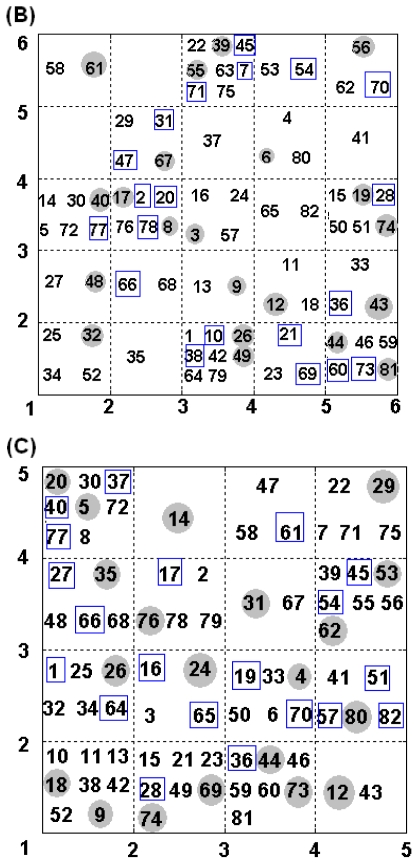
The distribution the 82 compounds in the 5 × 5 top-map of the Kohonen neural network: (**A**) is for the ERα set and (**B**) is for the ERβ set. (**C**) is the distribution of 81 compounds in the 4 × 4 top-map Kohonen neural network for the Selectivity set. Those numbers with grey circle background are compounds of the test set, while the others are the ones of the training set. Numbers in blue rectangles are compounds further split for the validation of the BRNN models.

Self-organizing maps are a special kind of neural network that can be used for clustering, visualization and abstraction tasks. SOM is especially suitable for data surveys because of its prominent visualization properties. We used a small Kohonen network with 5 × 5 = 25 neurons producing a map with 25 points for the ERα and ERβ sets, while for the Selectivity set, a map with 4 × 4 = 16 points was applied. The SOM built for all the data sets is shown in Figure 1. Compounds in the training and test sets, as well as the validation sets for the BRNN models are clearly marked.

### 3.1. ERα

#### 3.1.1. MLR

**Figure 2:**
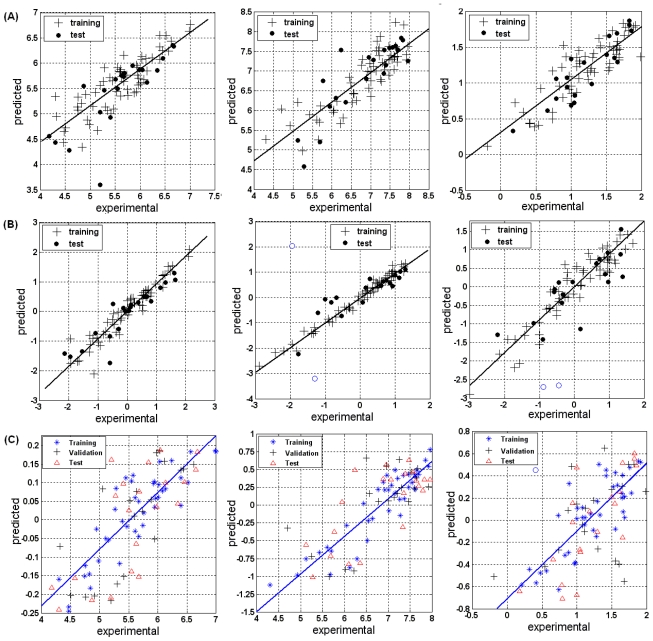
Experimental *vs* predicted pIC_50_ values of ligands for the ER alpha (left), ER beta (middle) and experimental vs predicted S values of ligands for Selectivity (right) by the MLR models (**A**) for the training and test sets, by the PLSR models (the pIC_50_ and descriptor values were normalized) (**B**). (**C**) Experimental and predicted values by Baysian-regularized neural network for the training, validation and independent test sets for ER alpha (left), ER beta (middle), and the Selectivity (right). The empty circles represent the outliers present.

A stepwise MLR method was employed to extract the descriptors most correlated with the relative bioactivity and the following optimal MLR model was arrived at:

(3) $$\begin{array}{l} pIC50=2.181(\pm 0.996)+44.287(\pm 11.238)\times JGI10-9.883(\pm 2.266)\times E1p\\ +2.998(\pm 0.397)\times R4u-0.577(\pm 0.090)\times BELTA96\\ {\text{n}}_{\text{tr}}=61,{\text{n}}_{\text{te}}=21,{\text{R}}^{2}=0.72,\text{SEE}=0.36,\text{F}=36.01,{\text{Q}}^{2}=0.63,\text{SEP}=0.44 \end{array}$$

where, n_tr_ and n_te_ are the number of compounds in the training set and the test set, respectively. R^2^ is the conventional correlation coefficient; Q^2^ is the external-validated correlation coefficient; F is the F-test value; SEE is the standard error of estimation for the training set; SEP is the standard error of prediction for independent test set. The experimental pIC_50_ values *versus* predicted pIC_50_ values are shown in Figure 2(A). From the figure, we can get the information the predicted pIC_50_ values for most of the compounds are well consistent with the experimental results, indicating the good performance of the built MLR model.

#### 3.1.2. PLSR

**Figure 3:**
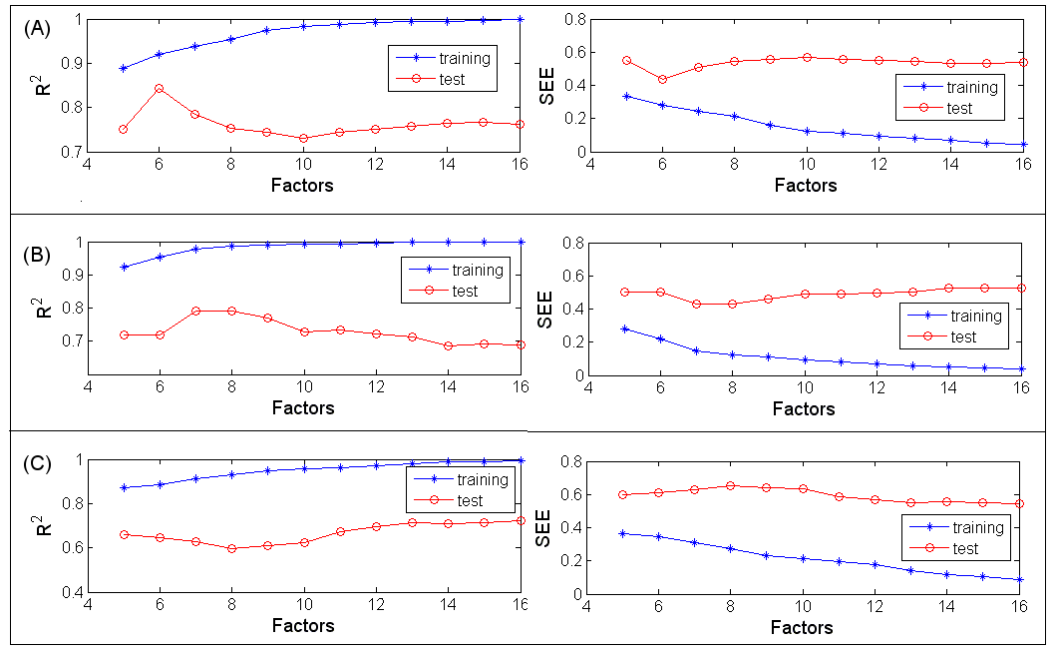
Trend of the statistical results of the PLSR models with vary latent factors based on the data sets for ER alpha (**A**), ER beta (**B**) and Selectivity (**C**).

PLSR is based on linear transition from a larger number of original descriptors to a small number of orthogonal factors (latent variables) providing the optimal linear model in terms of predictivity [3440]. All the variables were normalized before the PLSR procedure was taken by *x* = [*x* − *mea* (*x*)]/*std*(*x*). Herein x represents a variable and *std*(*x*) is the standard deviation. The resultant PLSR model with six latent variables outweighs others as shown in Figure 3(A). The corresponding statistical correlation coefficients (R_tr_^2^ and Q_te_^2^) are 0.92 and 0.84 respectively for the training and test set; while the Leave-One-Out (LOO) cross-validated coefficient of determination Q_cv_^2^ is 0.43. And the corresponding standard error is 0.28 for the model built and 0.44 for independent test data. The 6 latent factors totally explained 63.87% of the independent variances and 92.08% of the dependent variance. According to the Variable Importance in Projection (VIP), which summarizes the importance of X variables in the model, R4u, R4e, R5e, HATS5e, nPyridines, C-028, N-075, E1v, JGI10, E1p, R1e^+^ and RTe^+^ are ones among the most relevant descriptors. The experimental (normalized) *versus* predicted pIC_50_ values for both training and test sets were plotted in Figure 2(B). The model’s performance is good as most of the compounds are well distributed along the trend line.

#### 3.1.3. BRNN

The 61 compounds in the training set for the MLR and PLS models were further randomly split into one training set and one validation set with a ratio of 2:1 (Figure 1) for building the BRNN models. The simulation was iterated 50 times and the average predictive values were taken as the final result, in order to minimize the differences and random error. The optimal PCA-BRNN model has five hidden neurons, using five input neurons for the PCs, as displayed in Figure 2(C), with the statistical coefficient R_training_ is 0.87, R_validation_ is 0.76 and R_test_ is 0.73, while the sse(sum squared error) are 0.19, 0.09 and 0.10, for the training, internal validation and independent test sets, respectively.

#### 3.1.4. Surflex-Docking

**Figure 4:**
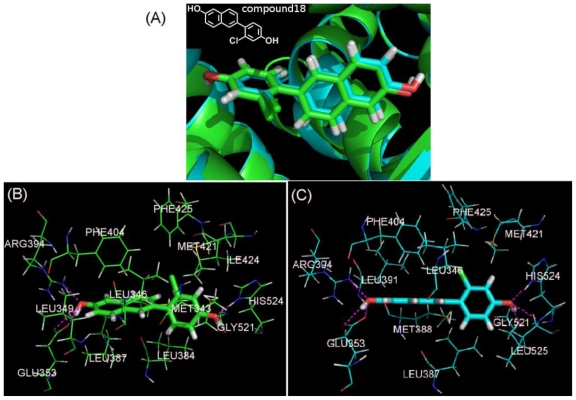
(**A**) Superpositon of Docking conformations of Compound 18 in 1X7R (green) and 1X7E (cyans).The interacting modes of compound18 with 1X7R (**B**) and 1X7E (**C**). Compound18 and the important residues for binding interaction are represented by stick and line models, respectively. The magentas dash lines denote the hydrogen bonds.

We implemented the docking process with prior minimized ligands and X-ray crystallographic data 1X7R and 1X7E retrieved from PDB. After running the Surflex-Dock, the scores of 10 docked conformers were ranked in a molecular spreadsheet. The crystallized ER structures with different resolutions and binding ligands greatly impact the docking accuracy and poses ranking. No precise correlation could be found between the top rank docking poses scores and pIC_50_ values when employing these two crystallized ER structures as the pIC_50_ values relate to number of events. In this study, we also docked the compounds into the crystallographic protein structures without water removing and no significant difference presents. In order to illustrate the interaction mechanism, compound18, the most potent ERα ligand among the 82 compounds, for more detailed analysis. Figure 4 generally represents the interacting model of compound18 with ERα when docked into 1X7R and 1X7E.

The binding conformation docked in the two crystal ERα structures are almost at the same position in the active site [Figure 4(A)] with the chloro substituent directing towards the hydrophobic group of PHE404, PHE425 and LEU346. As previous work proved, the hydroxyl of the phenyl ring has a H-bond with GLU353 and ARG394, and the hydroxyl of the naphthyl moiety may form a H-bond with HIS524 [9]. However, in our work, GlY521 and LEU525 can also form H-bond interactions with compound18, as shown in Figure 4(B),(C).

These H-bonds form the basis of the favorable binding interaction of the ligand with ERα. Still, the interactions caused by lipophilic features of the molecules play an important role in determining the binding affinity since a linear correlation between the ClogP and pIC_50_ was attained for these studied compounds (R = 0.32). Notably, the docking conformations of compound18 are totally different from the compounds bound to 1X7R and 1X7E (Table 2), as shown by their structural skeletons in the two X-ray structures. The binding orientations are different, but the H-bonding which plays a key role in the ligand-enzyme interaction are similar, *i.e.*, two similar H-bonds formed between the ligand with HIS524, GLU353 or ARG394. Therefore, the predicted conformation by this Docking method is reasonable.

### 3.2. ERβ

#### 3.2.1. MLR

Herein five descriptors were extracted, and with which the most predicative MLR models was built as shown below:

(4) $$\begin{array}{l} pIC50=41.527(\pm 5.532)+47.096(\pm 16.457)\times JGI10-19.060(\pm 3.445)\times E1p\\ -10.963(\pm 1.742)\times BEHe6-0.592(\pm 0.159)\times EEig09x+0.585(\pm 0.057)\times nCb-\\ {\text{n}}_{\text{tr}}=61,{\text{n}}_{\text{te}}=21,{\text{R}}^{2}=0.75,\text{SEE}=0.46,\text{F}=32.35,{\text{Q}}^{2}=0.75,\text{SEP}=0.46 \end{array}$$

Figure 2(A) shows the regression plot of experimental *vs* predicted *p*IC_50_ values of the compounds. All compounds in the test set are well distributed among the training ones, indicating the high quality of this model.

#### 3.2.2. PLSR

The optimal PLSR model was selected with seven latent factors, as shown in Figure 3(B) considering the reliability and predictive power, and totally explained 66.28% of the independent descriptors and 97.78% of the dependent variables. A plot of the experimental (normalized) *versus* predicted pIC_50_ values is shown in Figure 2(B); all the training compounds are well distributed along the trend line indicating its good performance (R_tr_^2^ is 0.98, SEE is 0.15, LOO Q_cv_^2^ is 0.28). When extrapolated to the test set, two compounds (compound44 and compound 61, marked as blue circles) were out of the application domain of the model. For the rest test set, the predictive capability is convincing, with Q_te_^2^ is 0.80 and SEP is 0.43. The importance of each descriptor was evaluated by VIP and the most relevant variables are nCb−, SP20, DP20, SP19, DP19, SP18, DP18, E1v, E1p, SP17, DP17, R4v, PJI2, nPyridines.

#### 3.2.3. BRNN

Same strategy was taken for the ERβ data set just as for the ERα data sets. Figure 2(C) presents the optimal BRNN models with five hidden neurons and 11 input neurons for PCs. The statistical results of the BRNN model are R_training_ is 0.91, R_validation_ is 0.70 and R_test_ = 0.74 with SSE_training_ is 0.29, SSE_validation_ is 0.14 with SSE_test_ is 0.15. No obvious overfitting can be observed from the model, and no outliers were considered. This suggests that the BRNN model can be applied to compensate for the deficiency of the linear models.

#### 3.2.4. Surflex-Dock

**Figure 5:**
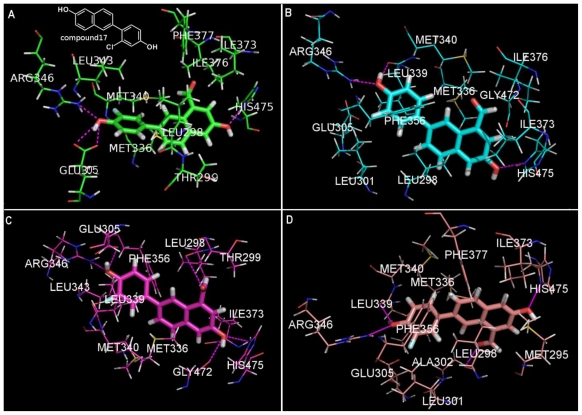
Compound17 and the potent interacting residues docking into 1QKM, 1X78, 1YY4 and 1YYE in sequence (**A–D**). The magenta dashed lines denote the hydrogen bonds.

Multiple crystals publicly available from the PDB were obtained and the binding mode of the studied compounds were generated in four crystals (1QKM, 1X78, 1YY4 and 1YYE). All the parameters were set as the default values in the whole process. The function scores were used to evaluate the binding qualities. Notably, docking into different crystal ERβ can have drastic effects on the ranking poses and relating scores. The first ranking pose scores failed to correlate pIC_50_ values precisely in each condition. The performance of docking for virtual screening and binding model investigation study should be cautiously analyzed. Figure 5 shows the putative binding mode of compound 17, the most potent ERβ ligand according to the experimental results, within the ligand binding pocket of the LBD of ERβ employing different crystal structures.

The main variation of the binding conformation is focused on the orientations of the formyl group attaching to the naphthyl part and the fluoro group attaching to the phenyl part. In the docking pose in 1QKM and 1YY4, the fluoro directs at MET340 and LEU343, while in 1X78 and 1YYE, the orientation of the phenyl group flipped and the fluoro substituent directs towards GLU305 and PHE 356. This further result in LEU339 instead of GLU305 forming a favorable H-bond with the hydroxyl group attaching to the phenyl part. Because of the flexibility of the rotaTable bond, the oxygen atom of formyl group orients obviously different. The docking conformations in 1YYE and 1YY4 indicate electrostatic interaction between the acyl of LEU298 and formyl group of compound17. While docking in 1QKM and 1X78, the formyl group is directed at MET336, ILE373 and ILE376.

### 3.3. Selectivity

Except for compound63 which showed the same binding affinity for both subtypes, the rest of the ligands studied herein tend to experimentally bind more in the LBD of ERβ. In order to study the contributing structural information, we defined the selectivity of binding affinity as showen by Equation 1 and developed MLR PLSR and BRNN models on the basis of compound 63 being excluded from the data set.

#### 3.3.1. MLR

A MLR model with R^2^ is 0.74 and SEE is 0.25 was reached (F = 25.56). When validated externally, the model well predicts all the compounds in the test set with Q^2^ is 0.80 and SEP is 0.21. Six molecular descriptors mostly correlating to the binding affinity property was selected as shown in Equation 5:

(5) $$\begin{array}{l} S=22.489(\pm 3.012)-7.709(\pm 1.107)\times BEHe6-2.602(\pm 0.552)\times BEHm5\\ +2.513(\pm 0.612)\times EEig03r-0.823(\pm 0.280)\times DISPe\\ -0.804(\pm 0.142)\times CIC2 \end{array}$$

A plot of the experimental and predicted pIC_50_ values is shown in Figure 2(A). The S value mainly ranges from 0 to 2. If S is zero the affinity capability of the corresponding compound for ERβ will be two-fold of that for ERα. Its increase strengthens the Selectivity between the two subtypes remarkably. Compounds gathered at the right top corner have strongest binding affinity to ERβ than to ERα. These are SERMs for ERβ that can be further studied and screened for drug design purposes.

#### 3.3.2. PLSR

A predictive QSAR model was produced using PLSR analysis to correlate variation in selective activity with variation in the descriptors. The optimum number of latent factors (six) corresponds to the highest correlation coefficient (R^2^ = 0.89) with the standard error of prediction is 0.35 [Figure 3(C)], while for LOO cross-validation, the correlation coefficient Q_cv_^2^ is 0.37. The predictive power was evalued by an independent test set. Compound18 and compound82 (genistein) were removed as outliers resulting in an improvement of Q_te_^2^ from 0.50 to 0.65 with a standard error of 0.61. The plot of experimental (normalized) and predicted S values for all compounds is shown in Figure 2(B). For the outliers (marked as blue circles) the predicted values are much lower than the experimental results.

#### 3.3.3. BRNN

The optimal BRNN model has five hidden neurons, with 14 input neurons for PCs. The performance of the BRNN model is not as good as that of the MLR and PLSR models and one outlier (compound57) was omitted from the validation set. For the training and test sets the conventional coefficients are 0.81 and 0.77, while for the validation set the cross-validation coefficient is 0.41. The resulting graphic model is provided in Figure 2(C). Compound57, marked with a blue circle, is obviously far away from the trend line and badly predicted. For all the BRNN models built for ERα, ERβ and Selectivity, the statistical results are summarized in Table 3.

#### 3.3.4. Docking Study

As mentioned above, no precise correlation could be found between pIC_50_ values and the first rank docking pose scores. It is not rational to investigate the ER subtype selectivity according to the docking rank pose scores. However, comparing the docking binding conformations could shed light on the possible contributing molecular properties that determining the Selectivity. Herein, we compared the binding conformations of compound22 and compound10 that have the highest ERβ subtype selectivity docked in 1X7R(ERα) *vs.* 1QKM(ERβ) and 1X78ERβ) *vs.* 1X7E(α).

**Figure 6:**
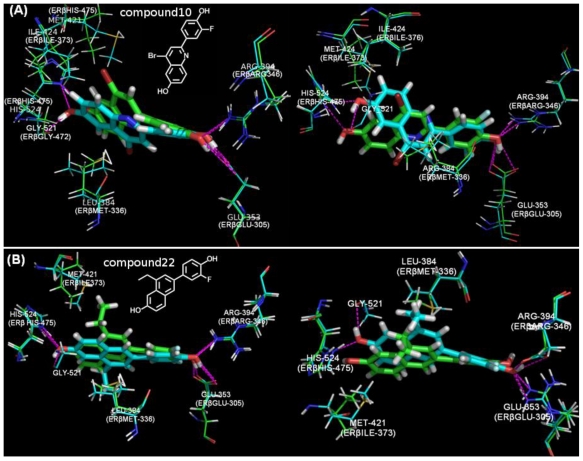
(**A**) Overlay of binding conformations of compound10 docking into 1QKM(ERβ) *vs.* 1X7R(ERα) (left) and 1X78(ERβ) *vs.* 1X7E(ERα) (right). (**B**) Overlay of binding conformations of compound22 docking into 1QKM(ERβ) *vs.* 1X7R(ERα) (left) and 1X78(ERβ) *vs.* 1X7E(ERα) (right). 1QKM and 1X78 were colored green, while 1X7R and1X7E colored cyan. The magenta dashed lines denote the hydrogen bonds.

Analysis of the X-ray co-crystal structures of ERα and ERβ complex with agonists illustrates that only two residue substitutions within 5Å expand the binding ligand: Met336 in ERβ replaces Leu 384 in ERα and Ile373 replaces Met421 [39]. However, orientation and conformations of the amino acids could obviously vary, such as ILE424 of crystal 1X7R and ILE376 shown in Figure 6(A). Similar H-bond interactions were found as docking in the ERα and ERβ crystals for the compounds studied. For some compounds HIS521 of ERα or HIS472 of ERβ could form H-bonds with the hydroxyl group of the naphthyl plane. However, this determines little in the binding affinity. We speculate that the H-bonding interaction is not the key factor determining the high selectivity between these two ER subtypes.

The most apparent difference when docking into ERα and ERβ is the naphthalene or the quinoline plane. The phenyl plane is inclined to adapt a similar space orientation and position in each crystal. The 8-ethyl substituent of compound22 and the 8-bromo substituent of compound10 directed towards ERβ Ile373 docking in 1QKM and 1X78, while docking into 1X7R and1X7E the 8-ethyl substituent of compound22 orients to Met421 or is rotated toward LEU384 and the 8-bromo substituent of compound10 was directed towards Met421 (Figure 6). These differences may explained by a favorable dispersive interaction with ERβ Ile373, relative to a less favorable interaction with ERα Met421 because of steric constraints of the ethyl group and the protein, or both. The importance of Met336 has been highlighted in determining the ERβ selectivity by other works [9,39,40]. These docking conformations of both compound22 and compound10 take the strategy that the naphthalene or the quinoline plane more apart from the Met336 than Leu384. Thus, we speculate that the naphthalene or the quinoline plane and the substituent in position 8 instead the hydrogen bond forms the most ER subtype selective pharmacophore.

## 4. Discussion

### 4.1 ERα Models

The MLR model was built with four descriptors. JGI10 is the mean topological charge index of order10, and the most important descriptors extracted that positively correlate with the binding affinity to ERα, which indicates the critical role of the overall charge dispersionh profile due to the influence of size and shape. E1p represents the 1st component accessibility directional WHIM index weighted by atomic polarizabilities. The atomic polarizability negatively contributes to the binding affinity. R4u, a GETAWAY descriptor, represents R autocorrelation of lag 4 (unweighted), demonstrates the positive effect of the molecule geometry and size and shape properties. BLTA96 is Verhaal model of algae base-line toxicity from MLOGP relating to the bind affinity of the lipotropism.

The PLSR model was built with six latent factors and successfully extrapolated to the independent test set. Thus, it can be used to further screen and discover new ERα ligands. According to the most important descriptors determined by VIP related to the MLR model, the importance of atom centred fragment types, interatomic distances and the shape of molecule with polarity and electronegativites are highlighted. The nitrogen of the quinoline and pyridine plane is a characteristic atom type correlating to the binding affinity to ERα.

The BRNN model was introduced as neural nets have the advantage of being able to explore nonlinear relationships between dependent and independent variables, even without prior knowledge of the form of the nonlinearity. In order to reduce the descriptor space and the chance of correlation among descriptors a principal component analysis was performed before the variables were used as the BRNN input data. The performance of the BRNN model compared to the MLR and PLSR models is not that good. The increasing PCs or hidden neurons did not improve the model quality inappreciably.

Comparing the models built, The PLSR model outperformed the others. The MLR model also successfully extrapolates to the independent test set. Here we recommend they could be applied to virtual screening of novel ERα ligands with improved affinity simultaneously to improve the accuracy.

### 4.2. ERβ Models

Five descriptors were selected for the MLR model built in this study. JGI10, the most positive correlated descriptor together with E1p, the most negative correlated descriptor, contributes to binding affinity to both ER alpha and beta isoforms. Another three descriptors correlate distinctively with the pIC_50_ for the ERβ: BEHe6 is the highest eigenvalue no. 6 of Burden matrix weighted atomic Sanderson electronegativities. nCb− is the number of substituted benzene. EEig09x is the eigenvalue09 from edges adjacency matrix weighted by edge degrees. These descriptors emphasize the importance of the molecule component structure characteristic.

Although two outliers (compound44 and compound61) were omitted when evaluated by the independent test set, the performance of the PLSR model is still considerable, with most of the compounds being tightly center around the trend line as shown in Figure 2(B). According to the VIP, the 10 most correlated descriptors mainly belong to Randic molecular profiles and most relate to the global molecular 3D structures and shape profile determined by atoms on molecular periphery, WHIM descriptors that elate to structure-property correlations atom-centred fragments, functional group counts and GETAWAY descriptors that are based on the row sums of the influence(distance)matrix. This indicates the importance of atom types of molecules and atoms on the molecular periphery, the distance between atom pairs and the electrotopological state of the functional group.

Compared with the MLR and PLSR models the BRNN model is not as powerful. 11 input neurons for PCs and five hidden neurons were used. For the test set compounds, the compounds are more dispersed from the trend line compared with the training and validation sets.

### 4.3. Selectivity Models

A total of 81 compounds were studied with the QSAR models. The optimal MLR model arrived at six descriptors: BEHe6, BEHm5 (highest eigenvalue n. 5 of Burden matrix weighted by atomic masses), EEig03r (eigenvalue 03 from edge adj. matrix weighted by resonance integrals), DISPe (d COMMA2 value weighted by atomic Sanderson electronegativities), CIC2 [complementary information content (neighborhood symmetry of 2-order)]. The graphical results visually indicate the performance of the PLSR model is comparable to that of the MLR model with six latent factors. The correlated descriptors determined by VIP give a deeper insight into the structural parameters which influence the pIC_50_ based ER subtype selectivity in comparison to the MLR model, RDF015m, BEHm6, H5p, E1p, RDF015e, nDB, HGM, BEHp8, L1m, H5v stress the importance of topological information and 3-D profiles, as well as the atom types and number of double bonds. Their definitions of all the descriptors can be found in the Dragon user manual and for the calculation details readers can refer to the Handbook of Molecule Descriptors [41].

One outlier (compound57) was detected in the validation set. However, its removal did not improve the predictive quality of the BRNN models when evaluated by the test set. Considering more input neurons for pcs and low Q_validation._, the BRNN failed to that accurately predict the Selectivity with the Dragon descriptors compared with the MLR and PLSR models, which would be of great help in screening ER subtype selective ligands.

### 4.4. The Docking Study

Docking method is an alternate to QSAR study in the drug screen and design procedure to discover and optimizing new ligands by predicting binding models and affinities of small ligands to biologically relevant target proteins. In this work, the Surflex docking method was implemented to understand the pharmacological preferences from the set of 2-arylnaphthalene and 2-arylquinoline derivatives. As a validation of the accuracy of the docking process, the RMSD of the crystal binding ligands from the crystals were compared with the top 10 ranked conformations redocked with Surflex-Dock. Before redocking, the ligands were minimized just like all the compounds studied. The results are summarized in Table 4.

Each of the energy minimized ligand exists 10 most possible conformations docked into the binding pocket of the ER crystals. The top ranked conformations corresponding Surflex Scores do not show precise correlation with pIC_50_ values. Besides the complexity of the pIC_50_ determinant factors, the binding conformation is influenced by multiple factors. Mikelos [42] has pointed out that the docking scores are highly sensitive to the source of ligand input conformations as small changes in the ligand input conformation can lead to large differences of the resulting docked poses. The energy minimized conformations were employed because the good performance of previous works studying QSAR and docking [40,43–46]. Differences between co-crystallized ligand proteins also lead to a large perturbation of the resulting docking performance as demonstrated by our study results above. It has been suggested that consensus scoring improves the enrichment of true positives.

However, this must be on the base that each individual scoring function is distinct and has relatively high quality [47]. It is unclear how the best docking pose could be selected. This results in the difficulty of using the Cscores to rank the docked poses. The highest Surflex-Dock scoring solution is supposed to be nearest to the experimental structure, but the RMSD analysis in Table 4 shows that the top ranking pose is not always the case. We suggest that the docking process used to screen and design the positive ER ligands with 2-arylnaphthalene and 2-arylquinoline scaffolds employ multiple crystallographic proteins, if available, and the results be comprehensively analyzed for each solution to greatly improve the accuracy. For example, in the docking of compound17 into 1YY4 in Figure 5, the top ranking pose confronts steric conflict as the fluoro group penetyrates into the protein too much, resulting in direct contact with LEU343. More importantly, the docking positive compounds should be further studied with the robust QSAR models in order to screen out the possible outliers and false positive compounds

## 5. Conclusions

This work has focused on the use of QSAR models and a docking program to study the molecular profiles most correlated with the binding affinity of estrogenic ligands and the origin of the ER subtype binding selectivity. MLR, PLSR and BRNN models were built respectively for the binding data for ERα and ERβ and the selectivity between ER subtypes via introduction of the S (selectivity) dependent endpoint. All the models were tested by an independent test set, which was not used for building the models for their prediction capability. JGI10 and E1p are the most correlated descriptors to binding affinity to both ER subtypes, while BEHe6, BEHm5 and EEig03r are especially vital in determining the selectivity according to the robust linear models. The use of multiple crystallographic proteins in the docking study should further improve the docking accuracy and be helpful for to the efficient identification of potential pharmacological groups. Hydrogen bond interactions form the base of the favorable interaction of ligands with both ERα and ERβ, but the binding affinity strength is more correlated with the atom fragment type, polarizabilities, electronegativites and hydrophobicity.

Compound22 and compound10 are the most ERβ selective compounds, as the docking results show that the spatial orientations of naphthalene or the quinoline plane and the substituent in position 8 are most correlated with the ER subtype selectivity. However, the top ranking pose scores failed to correlate precisely with pIC_50_ in each case with R^2^ < 0.2. Thus, it is difficult to determine the binding affinity of ER ligands only by the docking scores. Our results demonstrate the applicability and adaptability of the QSAR models and the necessity of performing docking processes using multiple crystallographic proteins to accurately screen and discover potential ER subtype selective ligands.

## Figures and Tables

**Table 1:** The SMILEs and pIC_50_ information of the compounds studied herein.

NO.	SMILES	pIC_50_(α)	pIC_50_(β)	S
compound1	OC1=CC=C(C2=CC(F)=C(C(Cl)=C(O)C=C3)C3=C2)C=C1	6.40	7.96	1.55
compound2	OC1=C(F)C=C(C2=CC=C(C=C(O)C=C3C#C)C3=C2)C=C1	6.14	7.92	1.78
compound3	OC1=C(F)C=C(C2=CC=C(C=C(O)C=C3F)C3=C2)C=C1	6.68	7.82	1.11
compound4	OC1=CC=C(C2=CC=C(C=C(O)C=C3C#N)C3=C2)C=C1	6.08	7.70	1.61
compound5	OC1=CC(F)=C(C2=CC=C(C=C(O)C=C3C#N)C3=C2)C(F)=C1	6.35	7.66	1.29
compound6	OC1=CC=C(C2=CC(C#N)=C(C=C(O)C=C3)C3=C2)C=C1	5.98	7.64	1.65
compound7	OC1=CC=C(C2=CC(CC)=C(C=C(O)C=C3)C3=C2)C=C1F	5.95	7.60	1.65
compound8	OC1=CC=C(C2=CC(C#N)=C(C=C(O)C=C3)C3=C2)C=C1F	5.68	7.57	1.89
compound9	OC1=CC=C(C2=CC=C3C(Cl)=C(O)C=CC3=C2)C(Cl)=C1	6.44	7.48	1.00
compound10	BrC2=CC(C3=CC=C(O)C(F)=C3)=NC1=CC=C(O)C=C12	5.55	7.47	1.92
compound11	BrC2=CC(C3=CC=C(O)C=C3)=NC1=CC=C(O)C=C12	5.67	7.37	1.68
compound12	ClC2=CC(C3=CC=C(O)C=C3)=NC1=CC=C(O)C=C12	5.67	7.34	1.66
compound13	ClC2=CC(C3=CC=C(O)C(F)=C3)=NC1=CC=C(O)C=C12	5.61	7.28	1.66
compound14	OC3=CC=C(C=C3F)C2=CC=C(C1=C2)C(C)=C(C=C1C#N)O	5.39	7.22	1.82
compound15	OC3=CC=C(C=C3)C2=CC=C1C(F)=C(C=CC1=C2)O	6.11	7.15	1.00
compound16	OC3=C(F)C=C(C=C3F)C2=CC=C1C=C(C=CC1=C2)O	6.04	7.08	1.00
compound17	OC1=C(C=C(C3=CC=C2C=C(O)C=C(C2=C3)C=O)C=C1)F	6.14	7.96	1.82
compound18	OC1=CC=C(C2=CC=C3C=C(O)C=CC3=C2)C(Cl)=C1	7.00	7.85	0.79
compound19	OC3=CC=C(C=C3)C2=CC(F)=C1C=C(C=CC1=C2)O	6.66	7.80	1.11
compound20	OC3=C(F)C=C(C=C3)C2=CC=C1C=C(C=C(C#N)C1=C2)O	6.02	7.68	1.65
compound21	OC3=CC=C(C=C3)C2=CC(Cl)=C1C=C(C=CC1=C2)O	6.52	7.64	1.08
compound22	OC3=CC=C(C=C3F)C2=CC(CC)=C1C=C(C=CC1=C2)O	5.63	7.62	1.99
compound23	OC3=CC=C(C=C3)C2=CC=C1C(Cl)=C(C=CC1=C2)O	6.04	7.60	1.55
compound24	OC3=C(F)C=C(C(F)=C3)C2=CC=C1C=C(C=CC1=C2)O	6.57	7.55	0.94
compound25	OC3=CC(F)=C(C(F)=C3)C2=CC=C1C(Cl)=C(C=CC1=C2)O	6.46	7.47	0.97
compound26	OC3=CC=C(C=C3F)C2=CC=C1C(Cl)=C(C=CC1=C2)O	5.84	7.40	1.54
compound27	OC3=C(F)C=C(C=C3)C2=CC=C1C(Br)=C(C=C(C#N)C1=C2)O	5.94	7.35	1.39
compound28	OC3=CC=C(C=C3F)C2=CC=C1C=C(C=CC1=C2)O	6.04	7.30	1.24
compound29	OC3=C(F)C=C(C=C3)C2=CC=C1C=C(C=C(C#CC)C1=C2)O	5.74	7.26	1.50
compound30	OC3=C(F)C=C(C(F)=C3)C2=CC(C#N)=C1C=C(C=CC1=C2)O	5.73	7.16	1.41
compound31	OC3=C(F)C=C(C=C3)C2=CC=C1C=C(C=C(C=C)C1=C2)O	5.28	7.14	1.85
compound32	OC3=C(F)C=C(C(F)=C3)C2=CC=C1C(Cl)=C(C=CC1=C2)O	5.93	7.07	1.11
compound33	OC3=CC=C(C(C)=C3)C2=CC=C1C=C(C=CC1=C2)O	6.40	7.00	0.48
compound34	OC3=C(F)C=C(C=C3F)C2=CC=C1C(Cl)=C(C=CC1=C2)O	5.28	6.97	1.68
compound35	OC3=CC=C(C=C3)C2=CC(C#N)=C1C(Br)=C(C=CC1=C2)O	5.88	6.92	1.00
compound36	OC3=CC=C(C=C3)C2=CC=C1C=C(C=CC1=C2)O	5.68	6.79	1.08
compound37	OC1=CC=C2C(C(C#N)=CC(C3=CC=C(O)C(F)=C3)=N2)=C1	4.98	6.64	1.65
compound38	OC3=CC=C(C=C3Cl)C2=CC=C1C(Cl)=C(C=CC1=C2)O	5.45	6.49	1.01
compound39	OC1=CC=C2C(C(C=C)=CC(C3=CC=C(O)C(F)=C3)=N2)=C1	5.41	6.36	0.89
compound40	OC3=C(F)C=C(C=C3F)C2=CC(C#N)=C1C=C(C=CC1=C2)O	5.26	6.24	0.93
compound41	OC1=CC=C2C(C(C#C)=CC(C3=CC=C(O)C=C3)=N2)=C1	4.82	6.12	1.28
compound42	OC1=CC=C2C(C=CC(C3=CC=C(O)C=C3)=N2)=C1Br	4.94	6.06	1.08
compound43	OC1=CC=C2C(C=CC(C3=CC=C(O)C=C3)=N2)=C1	4.75	5.77	0.97
compound44	OC1=CC=CC2=CC(C3=CC=CC(O)=C3)=CC=C12	4.84	5.69	0.78
compound45	OC1=CC=C2C(C(C(C)=O)=CC(C3=CC=C(O)C=C3)=N2)=C1	4.50	5.66	1.12
compound46	OC(C=CC2=C3)=CC2=CC=C3C1=CC=CC=C1	4.87	5.43	0.41
compound47	OC(C=CC2=C3)=CC2=C(C#CC)C=C3C1=CC=C(O)C(F)=C1	5.46	7.00	1.52
compound48	OC(C=CC2=C3)=C(Cl)C2=C(C#N)C=C3C1=CC=C(O)C(F)=C1	5.52	6.96	1.42
compound49	OC(C=CC2=C3)=C(Br)C2=CC=C3C1=CC=C(O)C=C1	5.58	6.89	1.29
compound50	OC(C=CC2=C3)=C(C)C2=CC=C3C1=CC=C(O)C=C1	5.55	6.77	1.19
compound51	OC1=CC=C2C(C=CC(C3=CC=C(O)C=C3)=N2)=C1	5.20	6.52	1.30
compound52	OC1=CC=C2C(C(Br)=CC(C3=CC(F)=C(O)C(F)=C3)=N2)=C1	5.11	6.44	1.32
compound53	OC1=CC=C2C(C(CC)=CC(C3=CC=C(O)C=C3)=N2)=C1	5.20	6.28	1.05
compound54	OC1=CC=C2C(C(C=C)=CC(C3=CC=C(O)C=C3)=N2)=C1	5.30	6.22	0.87
compound55	OC1=CC=C2C(C(CC)=CC(C3=CC=C(O)C(F)=C3)=N2)=C1	4.76	6.10	1.33
compound56	OC(C=CC2=C3)=C(OC)C2=CC=C3C1=CC=C(O)C=C1	5.05	5.94	0.83
compound57	OC(C=CC2=C3)=C( [N+]( [O−])=O)C2=CC=C3C1=CC=C(O)C=C1	5.15	5.70	0.41
compound58	OC1=CC=C2C(C(C4=CC=CC=C4)=CC(C3=CC=C(O)C(F)=C3)=N2)=C1	4.74	5.68	0.88
compound59	OC3=CC=C(C=C3)C2=CC=C1C=CC=CC1=C2	5.20	5.61	0.21
compound60	OC1=CC(C3=CC=C2C=CC(O)=CC2=C3)=CC=C1	4.58	5.25	0.56
compound61	OC3=CC=C(C=C3)C2=CC=C1C(C4=CC=CC=C4)=C(O)C=CC1=C2	4.91	5.13	−0.19
compound62	OC1=CC=C2C(C(OC)=CC(C3=CC=C(O)C=C3)=N2)=C1	4.18	4.92	0.66
compound63	OC1=CC=C2C(C(C(O)C)=CC(C3=CC=C(O)C(O)=C3)=N2)=C1	4.30	4.30	-
--mpound64	OC3=CC=C(C(F)=C3)C2=CC=C1C(Cl)=C(O)C=CC1=C2	6.24	7.92	1.68
compound65	OC3=CC(F)=C(C(F)=C3)C2=CC=C1C=C(O)C=CC1=C2	6.99	7.64	0.54
compound66	OC3=CC=C(C=C3)C2=CC=C1C(Cl)=C(O)C=C(C#N)C1=C2	6.01	7.52	1.50
compound67	OC3=CC=C(C=C3F)C2=CC(C=C)=C1C=C(O)C=CC1=C2	5.60	7.36	1.75
compound68	OC3=CC=C(C=C3)C2=CC(C#N)=C1C(Cl)=C(O)C=CC1=C2	5.96	7.22	1.23
compound69	OC3=CC=C(C=C3Cl)C2=CC=C1C=C(O)C=CC1=C2	5.97	6.96	0.94
compound70	OC3=CC=C(C(OC)=C3)C2=CC=C1C=C(O)C=CC1=C2	5.76	6.57	0.74
compound71	OC1=CC=C2C(C(C(C)=O)=CC(C3=CC=C(O)C(F)=C3)=N2)=C1	4.47	6.03	1.55
compound72	OC1=CC=C2C(C(C#C)=CC(C3=CC(F)=C(O)C(F)=C3)=N2)=C1	4.32	5.12	0.73
compound73	OC(C=CC2=C3)=CC2=CC=C3C1=CC=CC=C1O	4.30	4.70	0.18
compound74	OC(C=CC2=C3)=CC2=CC=C3C1=CC=C(O)C=C1F	6.62	7.70	1.04
compound75	OC(C=C(CC)C2=C3)=CC2=CC=C3C1=CC(F)=C(O)C=C1	5.95	7.60	1.65
compound76	OC(C=CC2=C3)=CC2=C(C=O)C=C3C1=CC=C(O)C(F)=C1	5.64	7.47	1.83
compound77	OC(C=CC2=C3)=C(F)C2=C(C#N)C=C3C1=CC=C(O)C(F)=C1	5.51	7.25	1.74
compound78	OC(C=CC2=C3)=CC2=C(C#C)C=C3C1=CC=C(O)C(F)=C1	5.61	7.20	1.58
compound79	OC(C=CC2=C3)=C(Cl)C2=CC=C3C1=CC=C(O)C=C1C	6.40	6.89	0.32
compound80	OC1=CC=C2C(C(C#N)=CC(C3=CC=C(O)C=C3)=N2)=C1	5.34	6.55	1.18
compound81	OC1=CC2=CC(C3=CC=CC=C3)=CC=C2C=C1	4.47	5.28	0.74
compound82	OC1=CC(O)=CC2=C1C(C(C3=CC=C(O)C=C3)=CO2)=O	5.40	7.01	1.60

**Table 2:** The crystals used in the docking process and 2D structures of their co-crystallized ligand.

Crystal	ligand	Crystal	ligand
**1X7R**		**1QKM**	
**1X7E**		**1X78**	
**1YYE**		**1YY4**	

**Table 3:** The statistical results of the BRNN models.

Data set	A*	B	R_training_	R_validation_	R_test_	SSE_training_	SSE_validation_	SSE_test_
**alpha**	5	5	0.87	0.76	0.73	0.19	0.09	0.10
**beta**	5	11	0.91	0.70	0.74	0.29	0.14	0.15
**Selectivity**	5	14	0.81	0.65	0.77	0.009	0.005	0.005

* A: represents the number of hidden neurons. B: represents the number of input neurons for PCs. SSE is abbreviation of Sum Squared error.

**Table 4:** Summary of the RMSD information when the cocrystalized ligand redocked into the corresponding crystals.

Crystal	AVG_RMSD	SD_RMSD	MAX_RMSD/NO. of pose	MIN_RMSD/NO. of pose
**1X7R**	0.66	0.18	0.94/7^th^*	0.46/10^th^
**1X7E**	0.32	0.03	0.36/9^th^, 10^th^	0.27/5^th^
**1QKM**	0.39	0.06	0.47/1^th^, 3^th^	0.32/8^th^
**1X78**	0.53	0.32	1.04/5^th^,7^th^	0.14/1^th^
**1YY4**	0.63	0.30	1.01/7^th^	0.14/6^th^
**1YYE**	0.77	0.54	1.81/7^th^	0.34/1^th^

* The numbers here match along with the ten plausible poses ranking with the docking score descending order.
